# Comparison of treatments for equine laryngeal hemiplegia using computational fluid dynamic analysis in an equine head model

**DOI:** 10.3389/fvets.2024.1478511

**Published:** 2024-12-24

**Authors:** Michelle L. Tucker, David G. Wilson, Donald J. Bergstrom, James L. Carmalt

**Affiliations:** ^1^Department of Large Animal Clinical Sciences, Western College of Veterinary Medicine, University of Saskatchewan, Saskatoon, SK, Canada; ^2^Department of Mechanical Engineering, University of Saskatchewan, Saskatoon, SK, Canada

**Keywords:** equine, CFD, computational fluid dynamics, laryngeal hemiplegia, recurrent laryngeal neuropathy, laryngoplasty, partial arytenoidectomy, respiratory-mechanics

## Abstract

**Introduction:**

Computational fluid dynamics (CFD) is gaining momentum as a useful mechanism for analyzing obstructive disorders and surgeries in humans and warrants further development for application in equine surgery. While advancements in procedures continue, much remains unknown about the specific impact that different surgeries have on obstructive airway disorders. The objective of this study was to apply CFD analysis to an equine head inhalation model replicating recurrent laryngeal neuropathy (RLN) and four surgical procedures. CFD was hypothesized to corroborate the order of the different trials based on impedance and to provide an impedance value numerically similar to the experimental results. In addition, it was hypothesized that CFD would offer insights into the changes in airflow associated with each procedure on a finite scale.

**Methods:**

An equine cadaver head underwent airflow testing and computed tomographic (CT) scans to replicate the disease state (RLN) and four surgical procedures: laryngoplasty, combined laryngoplasty and corniculectomy, corniculectomy, and partial arytenoidectomy. Pressure measurements at the pharynx and trachea were recorded, along with airflow data, for each trial.

**Results and discussion:**

The CFD and experimental models showed that partial arytenoidectomy had the lowest impedance in this case. While this procedure did have the largest rima glottidis area, the remaining procedural order was not dictated by the rima glottidis area. Recurrent laryngeal neuropathy and combined laryngoplasty with corniculectomy models showed negative pressure concentration on the luminal surface of the left arytenoid cartilage, which indicated a greater collapsing force on the tissue in this region. Narrowing within the caudal larynx at the level of the saccule showed increased negative pressure and higher velocity in the procedures with greater impedance, while partial arytenoidectomy exhibited more uniform pressure and velocity. Although this specific experimental head model contradicted previous flow studies, the CFD model reflected the experimental findings for the procedure with the least impedance and provided some insights into why these discrepancies occurred in this particular case.

## 1 Introduction

Equine upper airway surgery remains a complex challenge due to the nature of the diseases and the procedures used to treat them. Recurrent laryngeal neuropathy (RLN) can lead to significant airway collapse and is often treated with laryngoplasty (“tie-back”) or partial arytenoidectomy (PA) (1, 2). Both procedures were shown to improve impedance in an experiment with live horses, but the results are less consistent in *ex vivo* models (1, 3). As an alternative, arytenoid corniculectomy has been proposed, but it has only been explored in a vacuum flow model (4). Although these studies provided new information about these procedures, the understanding of fluid mechanics in the equine airway remains largely superficial and warrants further exploration to better represent the nuances of fluid mechanics in clinical patients.

Computational fluid dynamics (CFD) modeling continues to evolve as a useful diagnostic and decision-making tool in human respiratory surgery. It has been used to explore the relationship between complex human pharyngeal and laryngeal anatomy, vocalization, and obstruction (5–8). It estimates the development of negative pressure along the airway wall, which dictates the collapsing forces acting on the airway (9). The development of human CFD models has involved experimentation with various mesh types and turbulence models to determine the influence of these different techniques in a variety of applications (8, 10). Although airway CFD analysis continues to evolve, it has proven to be a powerful tool for identifying regions of greatest airflow resistance, anatomical variations that potentiate disease, and the ideal corrective surgical procedure on a patient-by-patient basis (7, 11, 12).

The use of CFD to evaluate surgical procedures in humans has provided insights into the interaction between surgical manipulations, airflow, and patient outcomes when addressing problems such as obstructive sleep apnea. CFD has been advocated as a pre-surgical decision-making tool to aid in the manipulation of airway geometry with the goal of decreasing resistance and improving airflow (7, 11). One study compared the effects of palatal stiffening, palatal resection, and mandibular advancement on pharyngeal collapse and demonstrated that all of these procedures improved airway mechanics in the patient geometry that was evaluated (7). This type of analysis has been performed for only one specific procedure in horses, establishing an “optimal” level of arytenoid abduction for laryngoplasty through the analysis of three different abduction levels.

One previously reported equine upper airway CFD model compared the differences between equine and human respiratory mechanics, further highlighting the complexity of equine respiratory mechanics (13). Although airflow during human breathing is primarily laminar/transitional and becomes turbulent only with heavy effort or obstruction, most equine respiration is overwhelmingly turbulent (13). Humans breathe roughly 12 times per minute with a tidal volume of approximately 0.5 L, while horses breathe an average of 10 times per minute with a tidal volume of 5.5 L. (1, 14, 15) Human airway cross-sectional areas range from 50 to 177 mm^2^, while the equine airway has been reported to range from 1,127 to 4,516 mm^2^ (14, 15). Reynolds numbers on the order of 100,000 have been reported based on flow parameters within equine airways; this indicates high turbulence compared to human flows, which have the Reynolds number ranging from 900 to 1,400 and are considered transitional (15, 16). Due to the complexity of turbulence in computational modeling, further development and validation of an equine CFD model are needed before it can be confidently applied to clinical patients.

A previous study examined the influence of RLN, left laryngoplasty with concurrent left ventriculocordectomy (LLP), laryngoplasty with left corniculectomy (LLPCOR), left arytenoid corniculectomy (COR), and PA on laryngeal impedance in an *ex vivo* model (4). The geometrical changes associated with each surgical procedure on the larynx were also examined (17). CFD was then performed to examine the flow patterns associated with each state within the larynx in the vacuum box model (18). The objective of this study was to investigate the influence of each procedure on flow development within the equine larynx *in situ*, using an experimental vacuum model of the entire equine upper airway, followed by CFD analysis of the model. The hypothesis was that the CFD model would confirm the experimentally derived results regarding the effects of surgical procedures on relative impedance. In addition, it was hypothesized that the changes in shape associated with each surgical procedure would influence the anticipated impedance of the larynx, the change in pressure across the larynx, and the amount of flow separation observed downstream from the larynx as air moves into the trachea. Finally, the development of negative pressure and wall stress within the larynx would be influenced by the upstream pharyngeal geometry, in contrast to the *ex vivo* model.

## 2 Materials and methods

### 2.1 Experimental data and image collection

**Figure 1:**
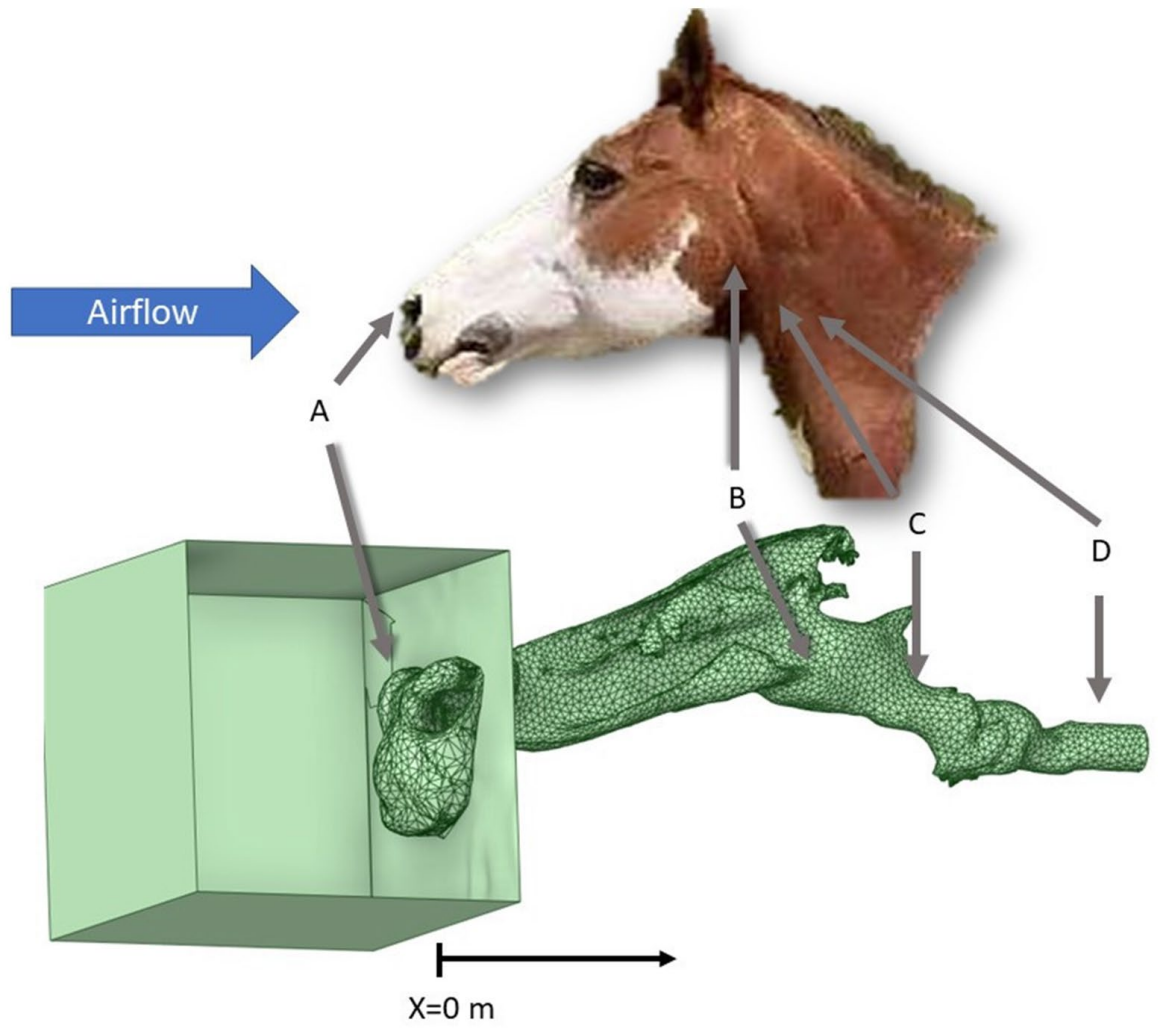
Final geometrical model with reference to the external view of a horse head. Point A represents the area just outside the nostrils where pressure is atmospheric and there is minimal velocity. Point B represents the area of the pharyngeal pressure measurement. Point C represents the area of surgical manipulation within the airway. Point D represents the area where the tracheal pressure measurement occurs, upstream from the orifice plate and vacuum.

One equine cadaver head from a six-year-old Quarter Horse mare with no history of airway disease was obtained postmortem, with the larynx *in situ* and four tracheal cartilage rings. The horse was humanely euthanized due to persistent septic arthritis and had no history or signs of airway disease. The head was kept on ice for approximately 14 h prior to testing. The horizontal portion of the mandible was removed to allow access to the soft palate by cutting through the lateral buccal cavity and performing bilateral osteotomies through the vertical ramus of the mandible. The soft palate and epiglottis were anchored using a #2 nylon suture (Ethilon, Ethicon US LLC, Bridgewater, NJ), passed through the cartilage and soft palate and tied to the hyoid apparatus, which was left intact. The nostrils were sutured in the open/flared position to replicate the muscular dilation that occurs during exertion. The pharyngeal region was observed endoscopically during this process and with airflow to determine whether collapse of the epiglottis or palate would occur during testing. The epiglottis was positioned using a suture through the base to the hyoid to replicate the ventral movement that occurs with head extension during maximal exercise in the live animal. The trachea was sealed over a section of polyvinyl chloride (PVC) pipe attached to a vacuum. The anatomy of the equine head is presented in Figure 1, along with the points of interest. An orifice plate was placed approximately 60 cm downstream from the tracheal outlet to measure airflow, and the vacuum was placed another 30 cm downstream. This was located on the right side of the head, as shown in Figure 1. This allowed air to be drawn through the head to replicate inhalation. Polyethylene tubing ran from a connector within the PVC pipe to a pressure transducer to measure pressure within the trachea relative to the room (Point D, Figure 1). An additional set of tubing and a transducer were used to measure pressure within the pharyngeal region by tunneling the tubing through a hole created with a 14-gauge needle and mosquito hemostats (Point B, Figure 1). Catheter placement in the pharynx was confirmed via endoscopic examination.

Five different scenarios were created in the upper airway at point C, as shown in Figure 1. The first replicated the disease state RLN, where the left arytenoid cartilage collapsed under airflow. Laryngoplasty was performed to replicate intact cricoarytenoideus dorsalis function on the right side of the larynx using a #5 Ethibond (Ethibond Excel, Ethicon US LLC, Bridgewater, NJ) suture, tied to a surgeon’s throw followed by four additional throws, while the left arytenoid was left to collapse into the airway. This was tested with airflow and induced tracheal pressure to replicate a horse with RLN. Next, left LLP was performed, as recommended as the standard treatment for RLN, using a laryngotomy approach to the ventricle with a roaring burr for exteriorization of the saccule (19). This was also tested with negative pressure generation. Left COR was then performed by removing the corniculate process of the left arytenoid cartilage, as reported previously (4). This was tested with the suture left intact, replicating combined LLPCOR. The left suture was then cut to allow the collapse of the arytenoid cartilage, replicating the plain COR procedure, and was tested. Finally, left PA was performed by excising the left arytenoid body and was tested (20).

Concurrent CT scans were performed to capture the three-dimensional geometry and anatomy of the head and airway under airflow conditions. The Toshiba Aquilon One (Canon Medical Systems, Markham, ON, CA) was used to acquire CT scans with 1 mm slices, employing a helical scanning algorithm. The scanning window could not extend from the nares to the trachea in one full run; however, the nostrils were scanned first, followed by the rest of the head and the nostril geometry. The nostril geometry was unified with the caudal anatomy during the geometry editing stage to create an entire head model.

### 2.2 Segmentation

The CT scans of the head for each state were segmented using Fiji (ImageJ, National Institutes of Health, Bethesda, MD), an open-source software used for generating three-dimensional views from DICOM images. Using the segmentation editor, the area of effective airflow was outlined slice by slice. The sinuses were not included in the model to reduce computational burden as previous studies have shown no significant airflow in the sinus regions (13). The slices were then compiled into an .stl file using the 3D viewer plugin and smoothed in MeshLab (MeshLab version 2020.12, Visual Computing Lab; CNR-ISTI, Pisa, Italy). Unconnected vertices and facets were removed using the corresponding filters, and the HC Laplacian smoothing and remeshing filters were applied as previously described (21, 22). The geometry was imported into Ansys® SpaceClaim (Ansys 2021 R1, ANSYS, Inc., Canonsburg, PA) for the manipulation and correction of irregularities to create watertight geometry. A box, approximately 200 mm^3^, was created in SpaceClaim to provide an inlet boundary away from the immediate nostril opening, and a nose imprint was created in the box to simulate inhalation in a static room of air. It was then imported into Fluent (Ansys 2021 R1 build 10,179, ANSYS, Inc., Canonsburg, PA) for meshing, and a surface mesh was generated. A volumetric mesh was then applied using a hexahedral mesh with approximately 10 million elements.

### 2.3 Numerical model

For the numerical model, the density, temperature, and humidity of air were held constant. The density of air was taken to be 1.204 kg/m^3^ at 20°C (15). Uniformity was assumed. Wall rigidity was also assumed, with the soft tissue in the fully developed flow state. This study aimed to replicate the airway during mid-inhalation to end inhalation, where the airway had accommodated the stress of negative pressure but airflow had not yet begun to decrease. Incompressible flow was also assumed.

The Reynolds-averaged Navier–Stokes (RANS) and continuity equations were solved as follows. Flow was assumed to be incompressible, isothermal, unsteady, and fully turbulent. As flow is primarily horizontal, the effects of gravity were considered negligible. The resulting Equations 1, 2, are presented below, as reported in previous models (15):


(1) $$\frac{\partial u_{j}}{\partial x_{j}}=0$$

(2) $$\frac{\partial u_{i}}{\partial t}+\frac{\partial u_{i}u_{j}}{\partial x_{j}}=-\frac{1}{\rho }\frac{\partial p}{\partial x_{i}}+\nu \frac{\partial^{2}u}{\partial x_{j}^{2}}-\frac{\partial \langle u_{j}^{\prime }u_{i}^{\prime }\rangle }{\delta x_{j}}$$

The *κ*-*ϵ* realizable turbulence model was used for the Reynolds stress, with standard wall functions applied in the near-wall region; a no-slip condition was assumed (13).

Ansys Fluent (Ansys 2021 R1 build 10179, ANSYS, Inc., Canonsburg, PA) was used to implement the finite volume method for solving the model equations. Convection was modeled using a second-order upwind differencing scheme. A SIMPLE scheme was then used to solve the pressure and velocity fields (15). The AMD Threadripper Pro 3955WX Processor (4.30 GHz) with 128 Gb of RAM was used to perform the simulations. Using 1,000 iterations resulted in decreased continuity residuals of 10^−2^ or less for each run, and it was observed that the residuals stopped changing. The average computation time for each simulation was approximately 1.5 h.

#### 2.3.1 Boundary conditions

The nostrils were each defined as inlets, the trachea was treated as an outlet, and the airway wall was defined as another boundary. Pressure within the trachea and atmospheric pressure outside the nares were used as the outlet and inlet pressures, respectively (Points D and A, Figure 1). The measured pressure within the trachea from each CT scan was used. As described above, this pressure was measured just downstream from the fourth tracheal ring for each laryngeal procedure, as shown in Figure 1.

#### 2.3.2 Mesh Independence study

A mesh independence study was conducted by generating a surface mesh with a local sizing of 0.5 mm in the region of the larynx and pharynx. A maximum mesh size of 20 mm was allowed, but this size was only reached in the box portion of the model. Five inflation layers were applied at the wall with a smooth transition. The maximum and minimum sizes were adjusted to generate mesh sizes of 1.7, 4.9, 6.9, 10, and 13.8 million cells, respectively, for the LLPCOR model. Planes of interest were generated parallel to the dorsal and axial planes, and the area-averaged pressure for each plane within each mesh size was calculated and plotted. The peak minimum pressure for each plane was also plotted. By examining the area-averaged pressure and peak minimum pressure for each plane, it was found that 10 million cells provided an appropriate balance between the anticipated calculation accuracy and computational demand.

### 2.4 Data analysis

Each procedural trial geometry was divided into cross-sectional planes, 4 mm apart, starting with the box edge as x = 0 m within the model. For each plane, pressure, velocity, and kinetic energy (as a measure of turbulence) were calculated as an area-weighted average. Within the pharyngeal and laryngeal regions, approximately x = 0.36–0.48 m, planes were added to create 1 mm spacing to better examine the changes specific to each procedure in that region. Trans-laryngeal impedance (I) was calculated by subtracting the measured experimental tracheal pressure from the area-averaged pharyngeal pressure at the catheter site calculated from the corresponding CFD model and divided by the volumetric flow rate, Q calculated at the CFD model outlet, as shown in Equation 3. For the experimentally reported impedance, the measured values for each of these variables was used.


(3) $$I=\frac{\left|P_{\text{pharynx}}-P_{\text{trachea}}\right|}{Q}$$

Separate planes were generated to perform the qualitative analysis. Planes parallel to the sagittal plane were generated, with a focus on the characteristics just inside the left and right sides of the larynx. Transverse planes were generated to examine the characteristics of the ventral larynx, mid-larynx, dorsal larynx, mid-pharynx, and mid-nostrils. Cross-sections were also captured at the mid-nostrils, mid-pharynx, laryngeal opening, mid-saccule, narrowest portion of the larynx, caudal dilation corresponding to the cricoid level, and finally a section at the level of the trachea. The larynx was specifically examined in the larger planes, with a focus on the region of the larynx to observe changes in pressure, velocity, and turbulent kinetic energy.

## 3 Results

The head model successfully replicated airflow in the equine upper airway, from the nares to the trachea.

### 3.1 Quantitative results

**Figure 2:**
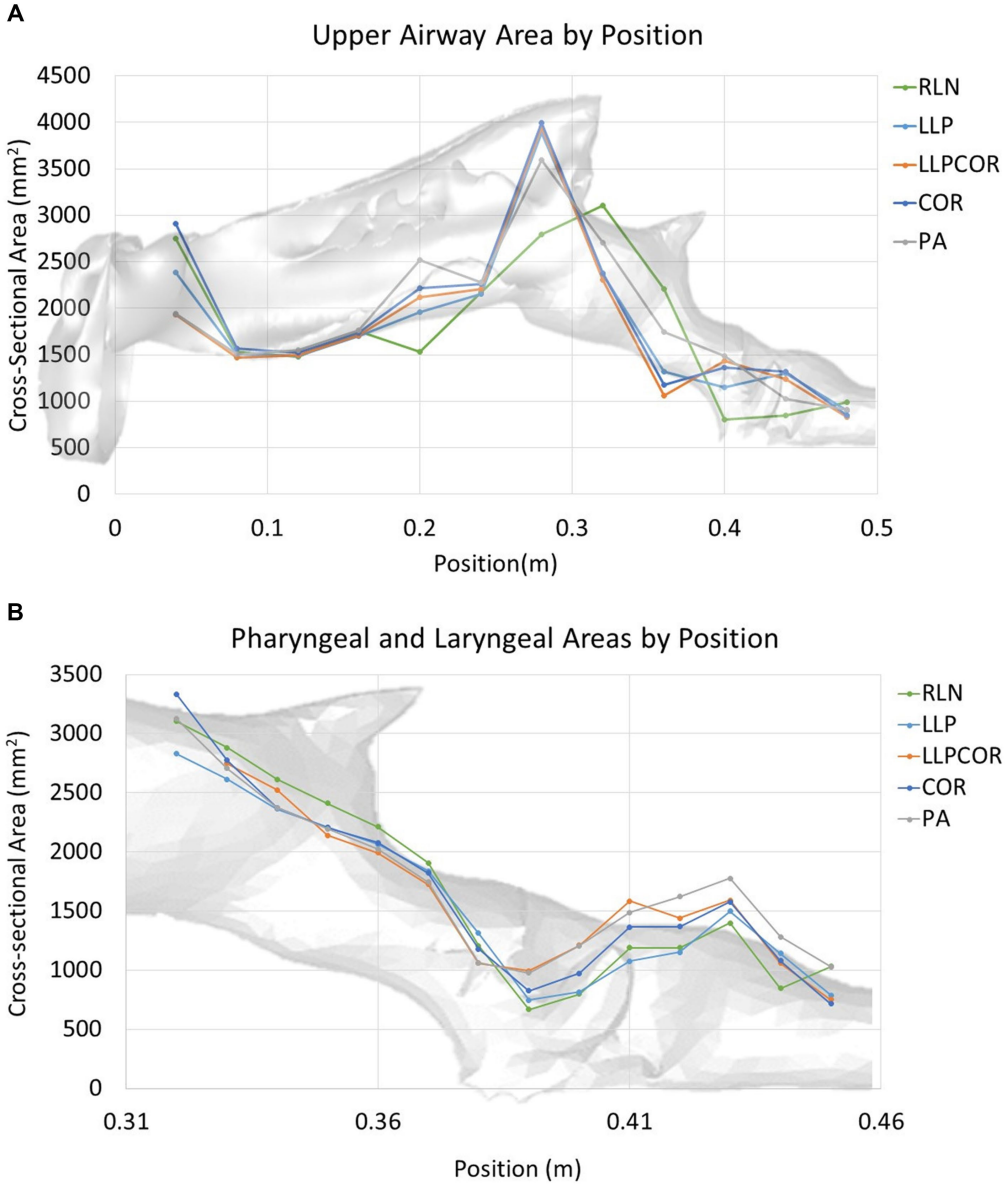
**(A)** Cross-sectional area along the upper airway by procedure. The RLN state exhibited the lowest cross-sectional area in the laryngeal region and showed the most variability across the cross-sectional areas in general. **(B)** Cross-sectional area along the larynx by procedure. The RLN state exhibited the lowest cross-sectional area at the region of the rima glottidis, while PA showed a larger area throughout the length of the laryngeal region. The pharyngeal region was similar between the procedures.

**Figure 3:**
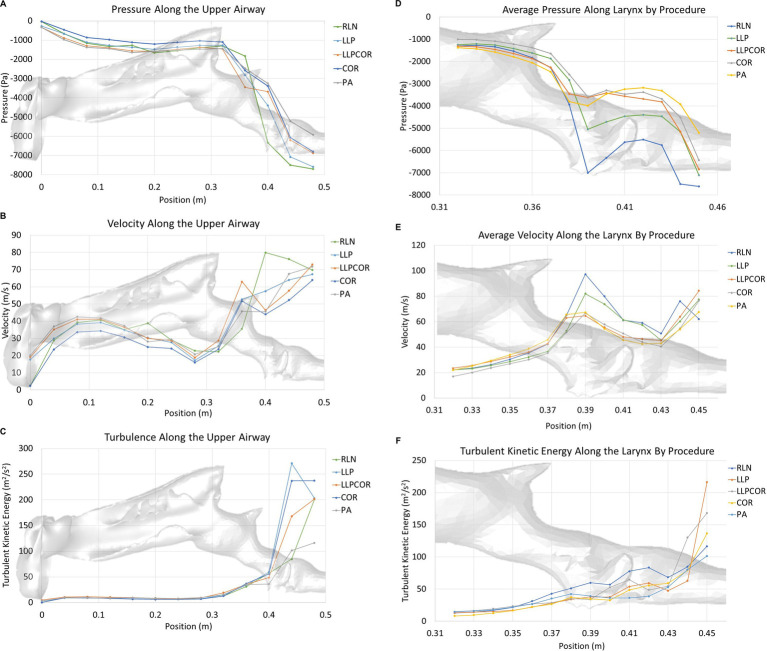
**(A)** Area-averaged pressure along the upper airway by procedure. The RLN state exhibited the lowest pressure in the region of the laryngeal opening, with LLP showing the second lowest pressure. LLPCOR, COR, and PA were more similar in their relative distribution. The LLPCOR procedure showed increased negative pressure in the caudal portion of the larynx as it transitioned into the trachea. **(B)** Area-averaged velocity along the upper airway by procedure. The RLN state showed the highest velocity in the region of the laryngeal opening, with LLP showing the second highest velocity. The remaining three procedures were more similar in their relative distribution. **(C)** Area-averaged turbulent kinetic energy distribution along the upper airway by procedure. Turbulent kinetic energy was largely similar across the procedures, except in the caudal laryngeal/tracheal region, where the cadaver model showed compression of the caudal airway. In general, the pharyngeal and laryngeal regions are the areas of increased turbulent kinetic energy compared to the rest of the airway. **(D)** Area-averaged pressure along the larynx by procedure. As demonstrated in the larger-scale plot, the RLN state exhibited the lowest pressure at the rima glottidis, best characterized as one significant pressure drop across that region. LLP showed the second-lowest pressure. LLPCOR, COR, and PA were more similar in their relative distribution. **(E)** Area-averaged velocity along the larynx by procedure. The RLN state exhibited the highest velocity along the larynx, followed by the LLP procedure. The remaining three procedures were more similar in their relative distribution. **(F)** Area-averaged turbulent kinetic energy distribution along the larynx by procedure. Turbulent kinetic energy specifically within the larynx was highest for the RLN state, with the LLPCOR procedure showing the second-highest level. All of the procedures showed increased turbulent kinetic energy at the laryngeal-tracheal junction.

Both the experimental and CFD scenarios identified PA as the procedure with the lowest impedance; however, the reported impedance was very different (83–87%) for all procedures. The measured tracheal pressure, pharyngeal pressure, flow rate, impedance, and rima glottidis area are reported in Table 1. The rima glottidis area was measured using the CT cross-sectional images and the final SpaceClaim® geometry. These areas are reported in Table 2. The LLPCOR procedure showed the largest difference in the rima glottidis area. Plots for the airway cross-sectional areas are shown in Figures 2A,B. Plots of pressure, velocity, and turbulent kinetic energy along the upper airway and specifically in the larynx are shown in Figures 3A–F.

**Table 1:** Experimental and computational (CFD) results for pharyngeal pressure (kPa), airflow (L/s), and impedance (kPa*s/L) by procedure.

		Experimental			CFD		
Simulated state	TP (Pa)	PP (Pa)	Airflow (L/s)	Impedance (kPa*s/L)	PP (Pa)	Flow rate (L/s)	Impedance (kPa*s/L)
RLN	−8,258	−563.5	10.8	0.712	−1379.5	57.7	0.1193
LLP	−8,409	−243.5	10.5	0.774	−1292.6	55.1	0.1292
LLPCOR	−7,500	−117.8	8.8	0.837	−1357.4	58.7	0.1046
COR	−7,319	−70.2	9.95	0.728	−1010.9	50.5	0.1249
PA	−7,106	−108.4	10.1	**0.695**	−1424.4	61.8	**0.0919**

Tracheal pressure was measured experimentally and used as the boundary condition for the tracheal outlet in the computational model. Note that the procedure with the lowest impedance in both instances was PA, highlighted in bold. CFD, Computational fluid dynamics results; TP, Tracheal air pressure; PP, Pharyngeal air pressure; RLN, Recurrent laryngeal neuropathy; LLP, Left-sided laryngoplasty with ipsilateral ventriculocordectomy; LLPCOR, Left-sided laryngoplasty with ipsilateral ventriculocordectomy combined with corniculectomy; COR, Corniculectomy; PA, Partial arytenoidectomy.

**Table 2:** Experimentally and computational (CFD) model rima glottidis area by procedure.

Rima Glottidis Area (mm^2^)			
Simulated state	Experimental	CFD	% difference
RLN	1,218	1,169	4.1
LLP	1,122	1,159	3.3
LLPCOR	1,482	1,314	11.4
COR	1,247	1,172	6.0
PA	1,671	1,593	4.6

The experimental rima glottidis area was measured from the corresponding CT section, while the CFD model rima glottidis area was measured using Space Claim needs a trademark. Percent difference refers to the difference between the experimentally measured and CFD model as compared to the experimental values. CFD, Computational fluid dynamics results; RLN, Recurrent laryngeal neuropathy; LLP, Left-sided laryngoplasty with ipsilateral ventriculocordectomy; LLPCOR, Left-sided laryngoplasty with ipsilateral ventriculocordectomy combined with corniculectomy; COR, Corniculectomy; PA, Partial arytenoidectomy.

### 3.2 Qualitative results

**Figure 4:**
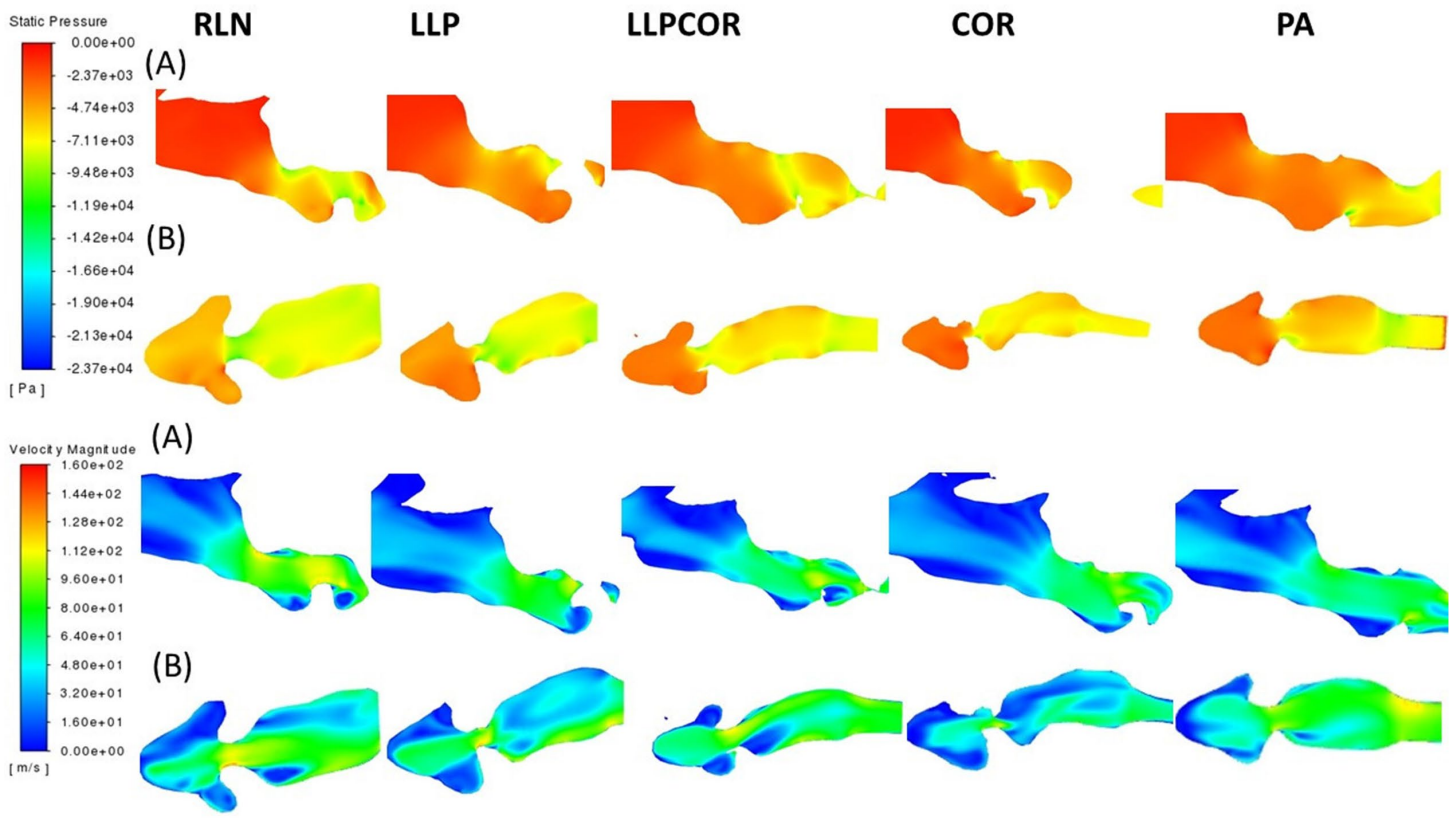
Pressure and velocity distribution through the left parasagittal and ventral laryngeal planes for all procedures. Row (A) shows the parasagittal left slice taken for each procedure, while row (B) shows the ventral laryngeal transverse section. Pressure is shown at the top, with velocity at the bottom. Air flows from left to right in the image, with the horse’s left side up in the transverse sections, the nostrils on the left, and the trachea on the right.

There were similar nostril and pharyngeal characteristics across the procedures. The parasagittal section observed just inside the left arytenoid cartilage showed higher negative pressure within the RLN state, LLP, LLPCOR, COR, and PA procedures compared to the right side. It was most pronounced for the RLN state. In the transverse planes, lower pressure and higher velocity were observed in the RLN state and LLP procedures. The LLPCOR, COR, and PA procedures appeared to be more uniform. All procedures showed a narrowed conformation ventrally caudal to the saccule, but this was more exaggerated in some procedures than in others. This is shown in Figure 4, where the top two rows of images show changes in pressure, while the bottom two show changes in velocity. Each of the (A) rows demonstrates the “lateral” view of the larynx, looking at a parasagittal plane captured just inside the left arytenoid cartilage, while the (B) rows demonstrate an orthogonal plane taken through the middle of the laryngeal ventricles dorsoventrally.

**Figure 5:**
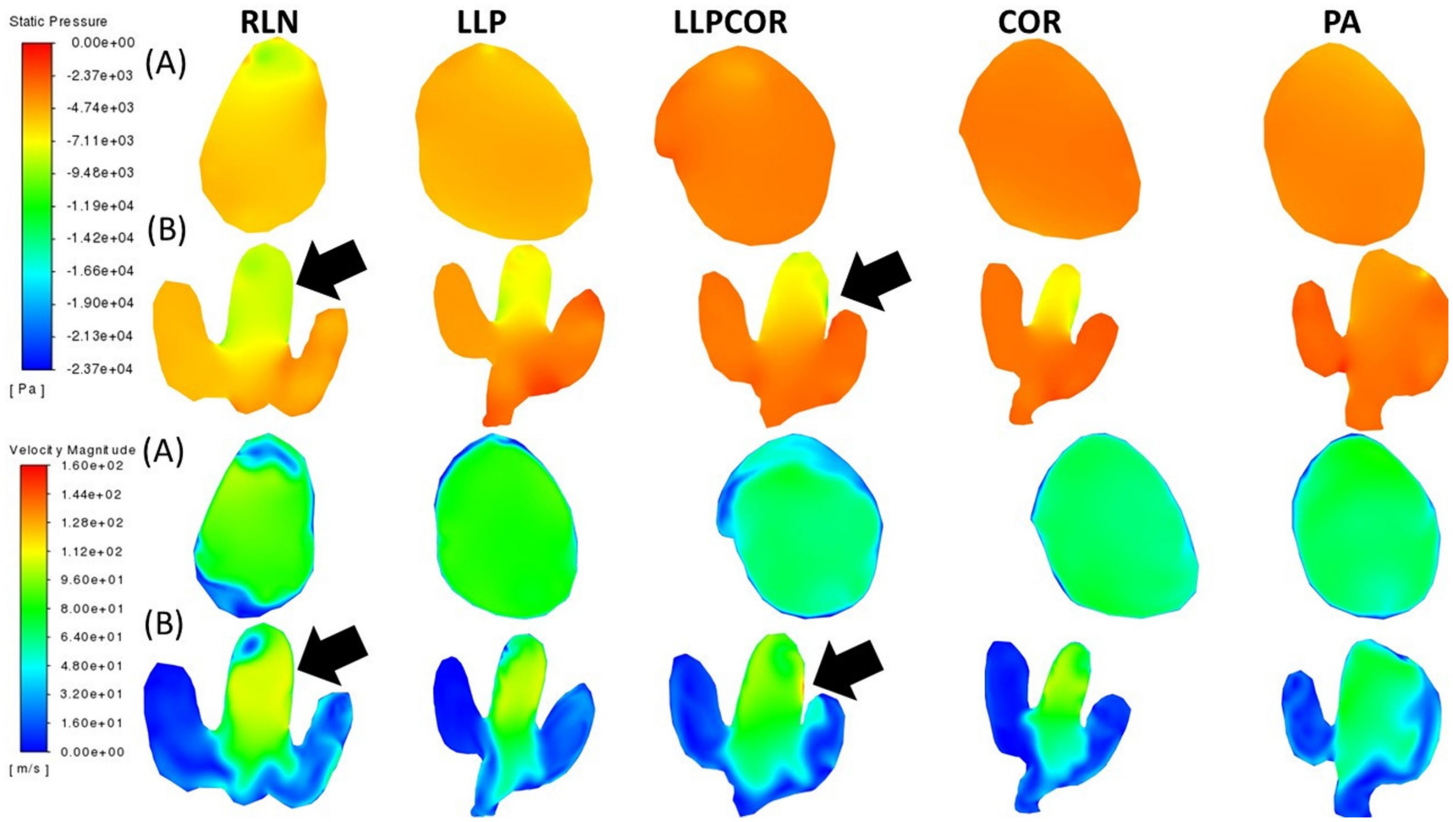
Laryngeal opening and mid-saccule cross-sectional planes by procedure. Row (A) shows the laryngeal opening slice taken for each procedure, while row (B) shows the mid-saccule section. Pressure is shown at the top, with velocity at the bottom. These sections are perpendicular to airflow and captured straight on so that the horse’s left side is on the right side of the image. The black arrows represent the regions of particularly low pressure or high velocity, observed with the RLN state and LLPCOR procedure.

The cross-sectional planes at the laryngeal opening showed narrowing for the RLN state, coinciding with more negative pressure and high velocity. At the mid-saccule region, greater negative pressure was observed inside the left wall of the RLN state and LLPCOR procedure models, denoted by black arrows in Figure 5. The top two rows display the pressure distributions within the larynx, while the bottom two display the velocity distributions. The removal of the arytenoid body in the PA procedure expanded the region on the left side; and therefore, decreased air velocity and less negative pressure were observed. Even caudal to the saccule, there was a similar trend of less negative pressure and reduced velocity in the narrowest portion of the PA procedure. These are shown in Figure 5, where row A depicts the laryngeal opening and row B shows the opening of the ventricles.

All of the planes captured and examined can be found in the Supplementary Information. Pressure, velocity, and turbulent kinetic energy were all examined in cross-sectional, sagittal, and transverse planes. All were adjusted to the same color scale for each of the planes, as reported by the procedure. The differences in the shape of the planes were due to the position of the head within the CT.

## 4 Discussion

The experimental and CFD models were in agreement regarding the procedure with the lowest impedance for this horse model but had slight differences regarding the overall procedure order. The numerical comparison showed a larger disparity between the impedance values for the laryngeal region in the experimental and computational values than what was expected based on previously reported equine and human models (13, 15, 23). The flow rates reported from the experimental portion of the study were lower than expected. This discrepancy may have resulted from a measurement error due to small-scale oscillations in the flow, caused by passage through the tissue, which could have disrupted the orifice plate measurements. Alternatively, it could reflect an accurate representation of collapse within the cadaver. The CFD model reported values that were much closer to the anticipated values for the flow rate and therefore might have been more accurate in this case.

High impedance was reported between the pharynx and the tracheal outlet, both numerically and relative to the impedance at different portions of the airway. Although multiple studies have reported that 50% of the upper airway resistance should be attributable to the nasal passage, this was not the case in the present study. A number of factors may explain this discrepancy (13, 24, 25). Throughout each of these studies, different breeds were used. In this study, a Quarter horse was used as the model, while a previous study used a Thoroughbred (13). It is also important to consider the application as the current study sought to establish a truly inhalation-based model by subjecting the head to negative airflow after stabilizing the collapsible portions of the airway. This approach might have resulted in higher reported impedance in the regions of the collapsible tissue. Rakesh et al. (13) also found a constriction in the caudal nasal region where air dropped in the pharynx, which resulted in high negative pressure and turbulence. The horse used in this study did not have the same narrowed region, and even the original CT images showed significant overlap between the caudal nasal passage and the pharynx in the current study model. Some of the earlier studies reporting higher nasal resistance also incorporated transducer catheter placement in the pharynx but did not specify the exact location within that region. Pharyngeal resistance varies significantly from rostral to caudal, which could influence the reported resistance and explain the disparities between studies (24, 26). The current study correlated more closely with a previous fiberglass model, which found the nares, pharynx, and larynx each contributed approximately 30% of the airway resistance (25). In addition, the negative tracheal pressure was much higher than in some reports. However, it has been demonstrated that horses compensate for upper airway resistance by generating more negative pressure in the lower airways, within the ranges reported here for tracheal pressure (27).

This study followed a similar approach to a previous study, wherein the equine larynx was modeled in isolation (18). A similar jet effect from the caudal nostrils into the larynx was observed, as seen before. However, the anatomical change in direction ventrally from the ethmoid region, dropping into the pharynx and then subsequently into the larynx, was much more abrupt than in the straight box model. The funneling effect of the pharynx was observed, as documented previously (18). There appeared to be less abrupt changes in the airway, which might have been a function of the geometry construction. In this study, airflow regions were traced manually to establish the geometry, while in the previous study, the tissues were isolated. Manual segmentation was necessary in this case as the shape/contour of the sinuses and conchal bullae were expected to confound the process of semi-automated segmentation. Across both studies, the RLN state and COR procedure were more obstructive and characterized by higher negative pressure and velocity. The saccule also influenced the flow, as shown in Figure 4. There was a pattern of sudden changes in pressure and velocity over a short distance with the higher impedance procedures, as noted in the other study. Unique to this study, the LLP and LLPCOR procedures did not reduce impedance as effectively and the PA procedure exhibited the lowest impedance. One explanation for this is that the head was oriented in the upright position, which might have resulted in the compression of the ventral soft tissue structures and reduced airway space. The PA procedure might have resulted in a greater cross-sectional area in this region through simple tissue excision alone. The use of a cadaver head resulted in unexpected impedance findings, with the LLP procedure showing higher impedance than the RLN state. Narrowing of the airways at the level of the nostrils and in other regions might have contributed to these unexpected results. The use of live patient geometry would be beneficial for future studies. In addition, the inclusion of more study subjects is warranted.

One important outcome of this study, which is supported by other studies, is that the PA procedure did not have the lowest velocity or the highest negative pressure, despite having the lowest impedance. Looking at the rima glottidis region alone, the negative pressure for the PA procedure was not the highest. However, upon proceeding caudally, the PA procedure compensated in the more caudal regions of the larynx, especially as the left ventricle region was incorporated into the cross-sectional flow area. This aligns with the modified PA report, which indicated that the changes in the rima glottidis area did not reflect the changes in impedance observed with the LLP and PA procedures (1). Looking at one specific portion of the airway is not entirely predictive of impedance, and the use of CFD allows for the incorporation of the entire airway (18). This finding is also supported by multiple sleep apnea studies in humans, where airway volume was correlated with impedance (5, 28).

One significant limitation of this study is the use of a single subject in the creation of the CFD models for the various surgeries, meaning that the relative outcomes regarding the different surgical procedures may only apply to this horse. However, one of the goals of this preliminary investigation was to determine whether a CFD model could corroborate the experimental “fitness” of a particular surgery within an individual “patient,” which was successfully achieved. The findings regarding the differences between the surgical procedures and flow characteristics should be interpreted with caution as this model is one of only four equine-based CFD models reported in the literature to date (13, 15, 18, 22). Another limitation is the use of cadaver tissue instead of live tissue. The use of a cadaver was considered more ethical and potentially representative as the alternative— using a horse under anesthesia—would still have presented similar problems with airway soft tissue collapse, as observed in this study. It was decided to use fresh tissue preserved on ice due to concerns about the consistent freezing and thawing of a cadaver head, with special consideration for the respiratory mucosa and cartilages.

Although the experimental portion of the study reported the same findings for airflow and impedance as reported in other studies, the CFD results were much closer to the expected findings based on previous literature and offered insights into why the experimental discrepancy potentially occurred. The differences in nasopharyngeal geometry between this equine patient and a previous study, along with the consistent expansion over distance provided by the PA procedure and the consistent pressure and velocity throughout the volume, were all more apparent in the CFD analysis and help explain the disparities. Capturing an accurate representation of the three-dimensional upper respiratory anatomy in the equine patient is a worthwhile pursuit, given the potential insights that can be gained from the advanced analysis provided by CFD. This study is unique to both the veterinary and human fields in seeking the validation of a CFD model based on experimental data obtained from a cadaveric model. Further calibration and refinement of CFD application in an equine head model are necessary before it can be applied to clinical patients. However, the potential benefits are significant, given the ongoing advancements in computational and mechanical knowledge.

## Data Availability

The original contributions presented in the study are included in the article/Supplementary material, further inquiries can be directed to the corresponding author.

## Supplementary material

The Supplementary material for this article can befound online at: https://www.frontiersin.org/articles/10.3389/fvets.2024.1478511/full#supplementary-material.
