# Supplementary material for: Comparison of treatments for equine laryngeal hemiplegia using computational fluid dynamic analysis in an equine head model
Source: Front Vet Sci. 2024 Dec 24;11:1478511. doi: 10.3389/fvets.2024.1478511 (PMC11703841; doi:10.3389/fvets.2024.1478511)

Supplemental Information

The slices that were collected and analyzed as referenced in the manuscript are included here. Images have been modified to fit on the page.

Figure A Pressure (top row), velocity (middle row), and turbulent kinetic energy (bottom row) for the nostril cross-sections by procedure.

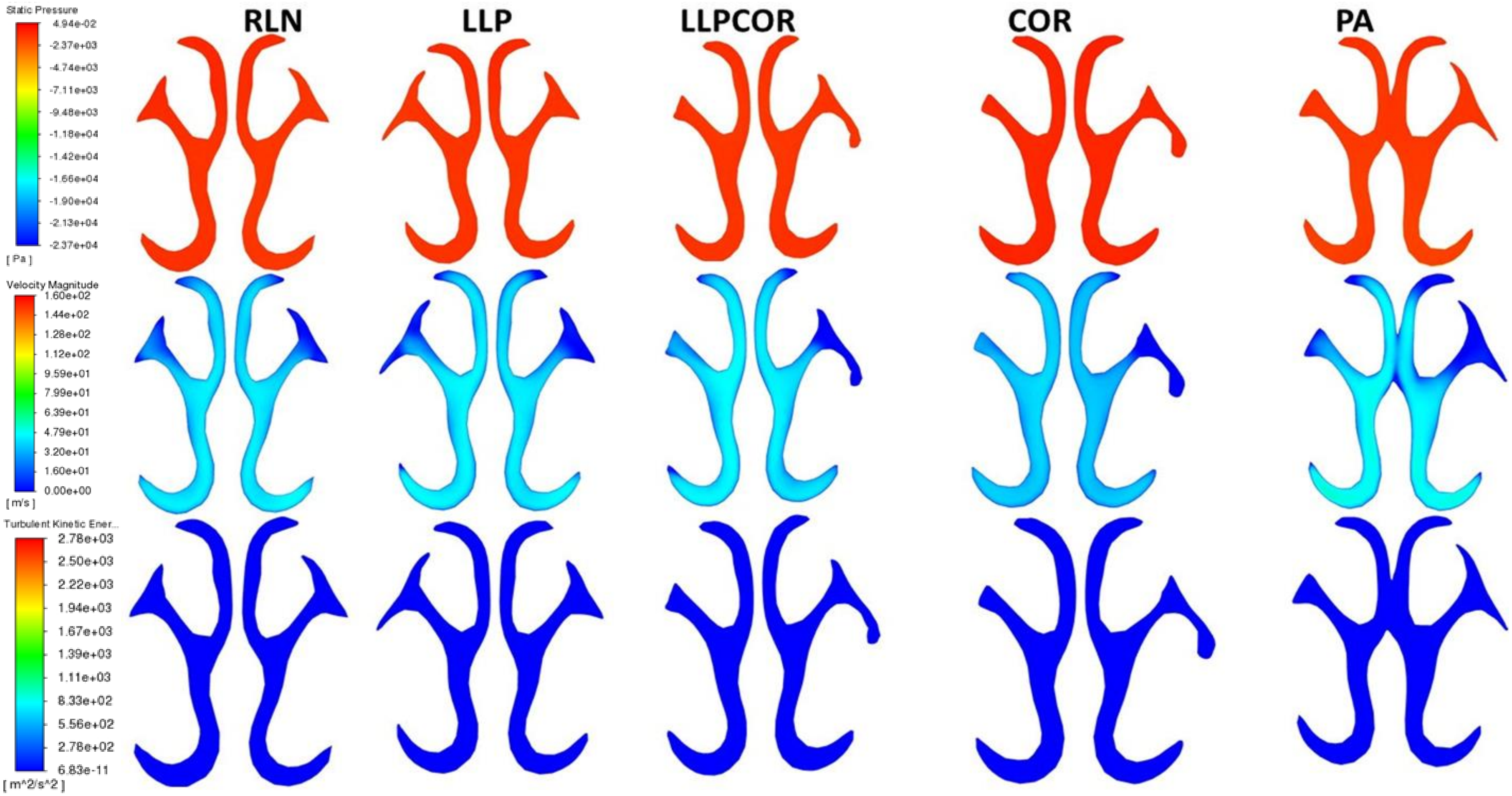

Figure B Pressure (top row), velocity (middle row), and turbulent kinetic energy (bottom row) for the mid-pharyngeal cross-sections by procedure.

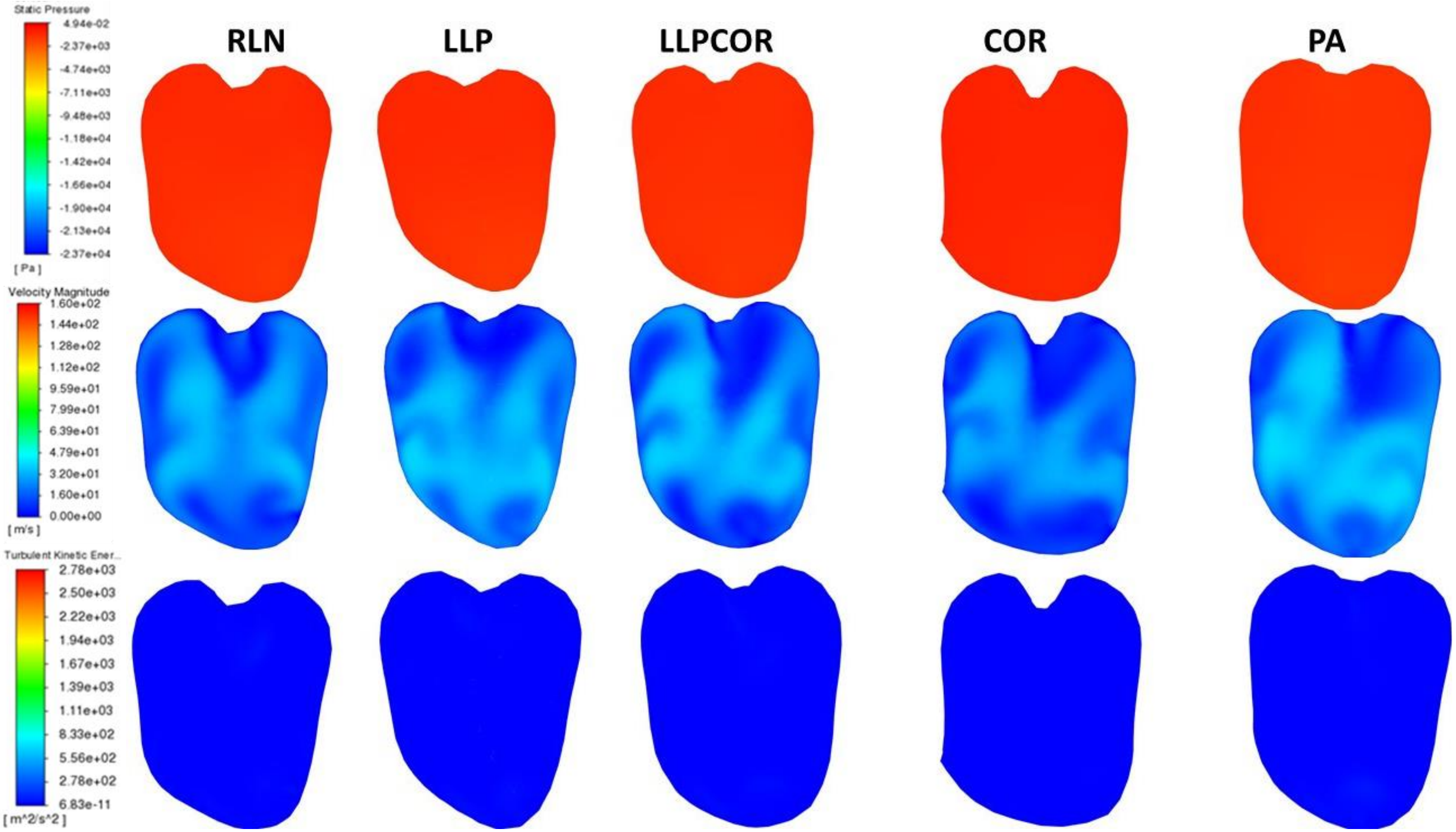

Figure C Pressure (top row), velocity (middle row), and turbulent kinetic energy (bottom row) for the laryngeal opening cross-sections by procedure.

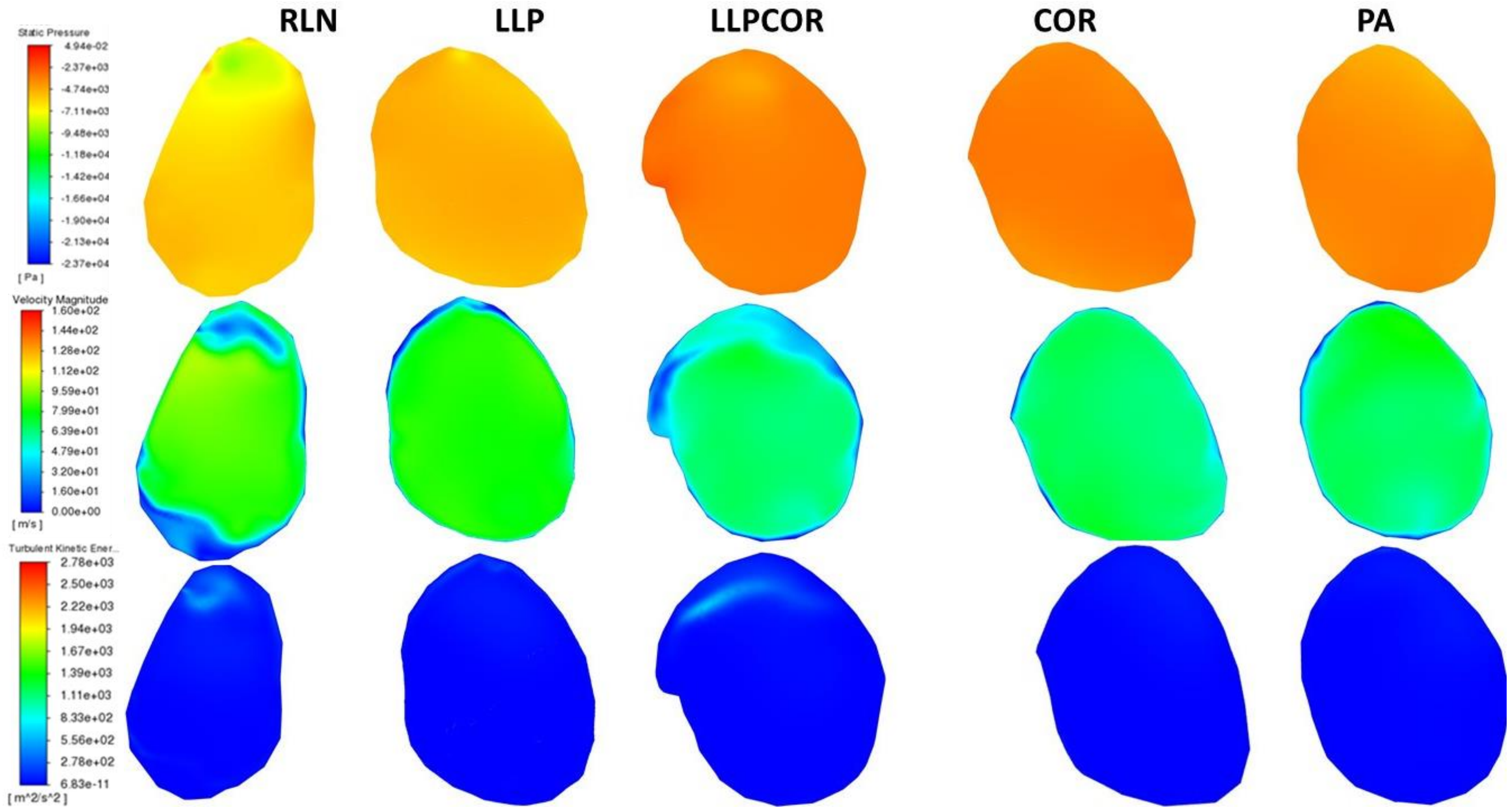

Figure D Pressure (top row), velocity (middle row), and turbulent kinetic energy (bottom row) for the mid-saccule laryngeal cross-sections by procedure.

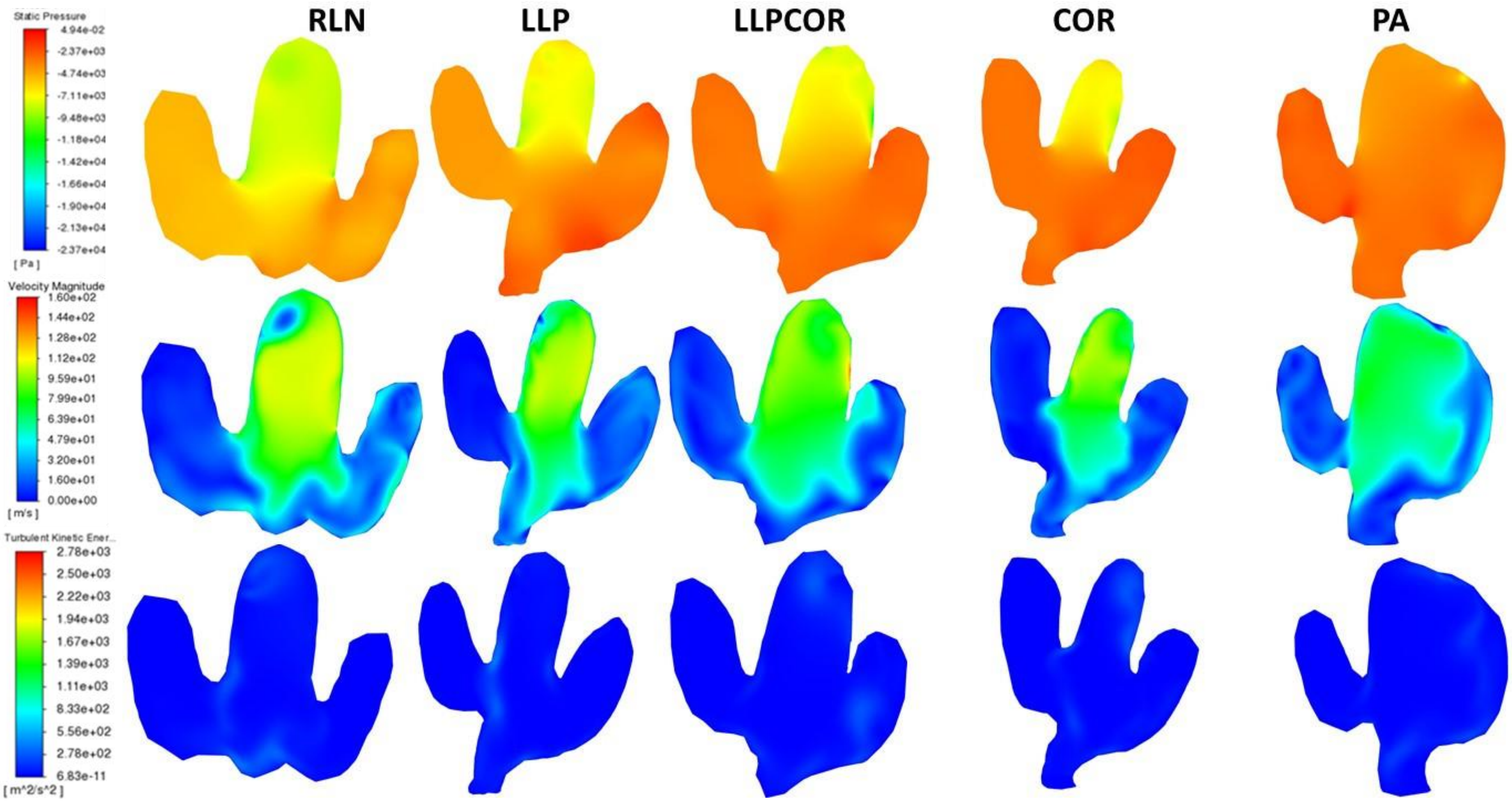

Figure E Pressure (top row), velocity (middle row), and turbulent kinetic energy (bottom row) for the narrowest laryngeal cross-sections by procedure.

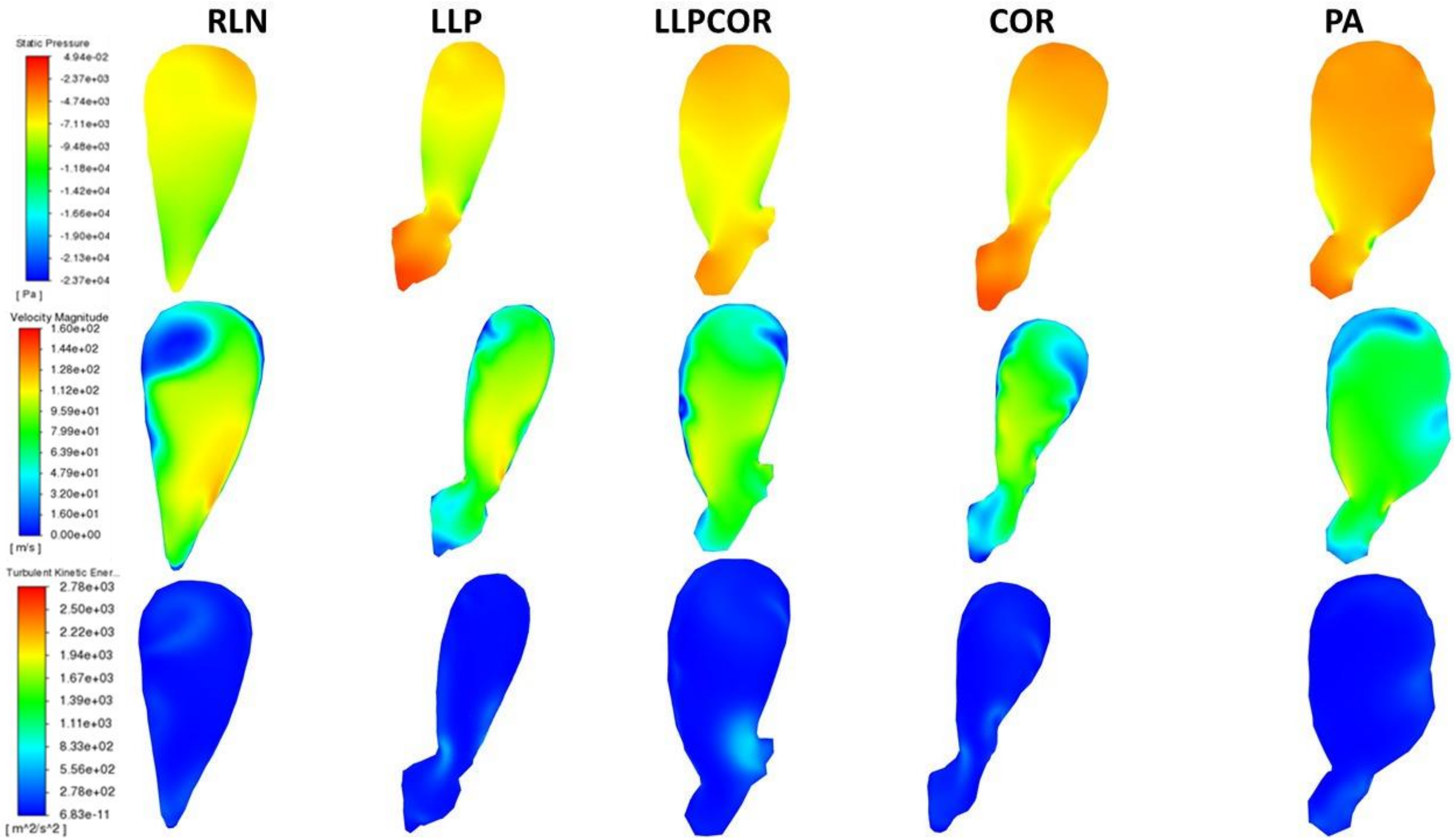

Figure F Pressure (top row), velocity (middle row), and turbulent kinetic energy (bottom row) for the caudal laryngeal cross-sections by procedure.

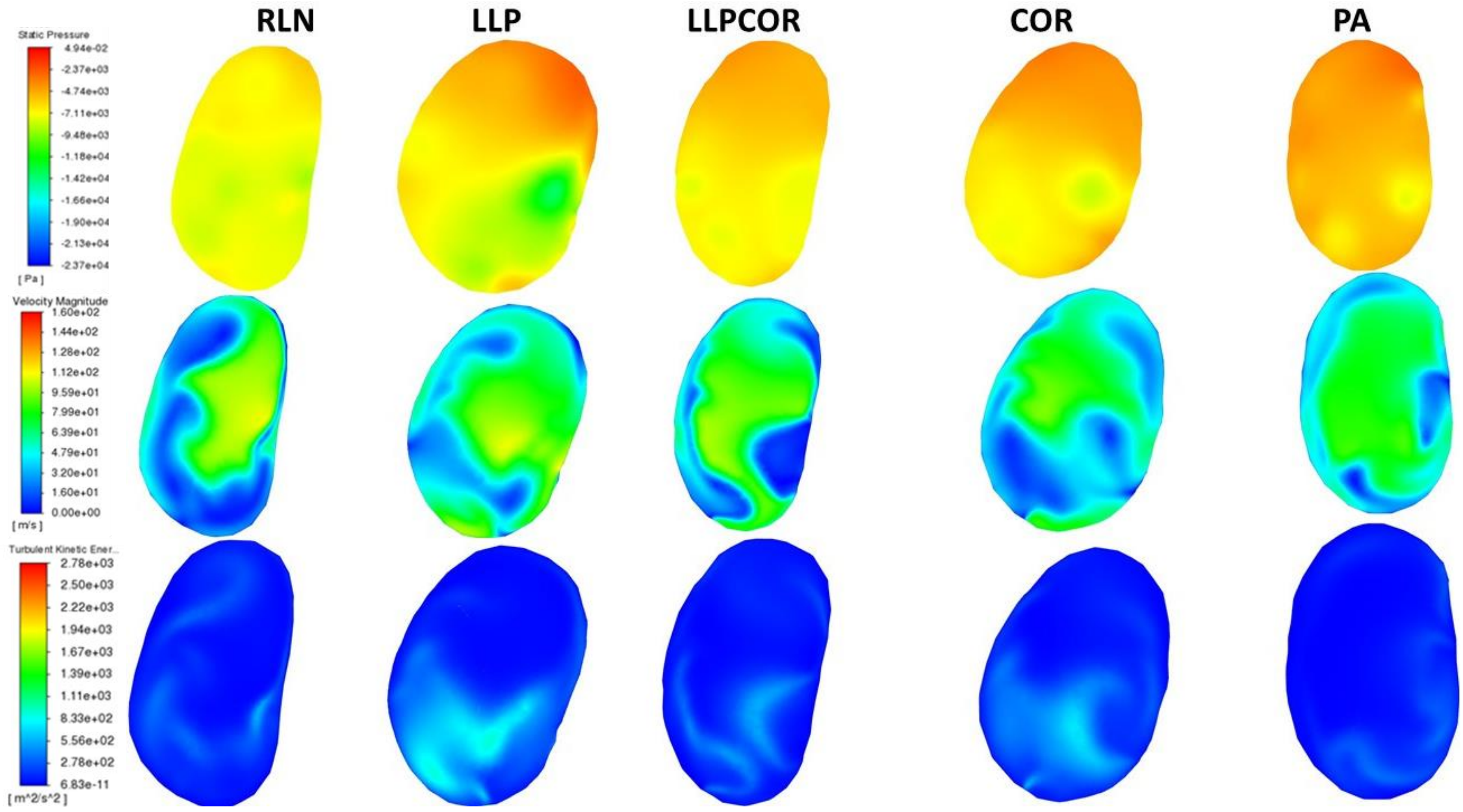

Figure G Pressure (top row), velocity (middle row), and turbulent kinetic energy (bottom row) for the tracheal cross-sections by procedure.

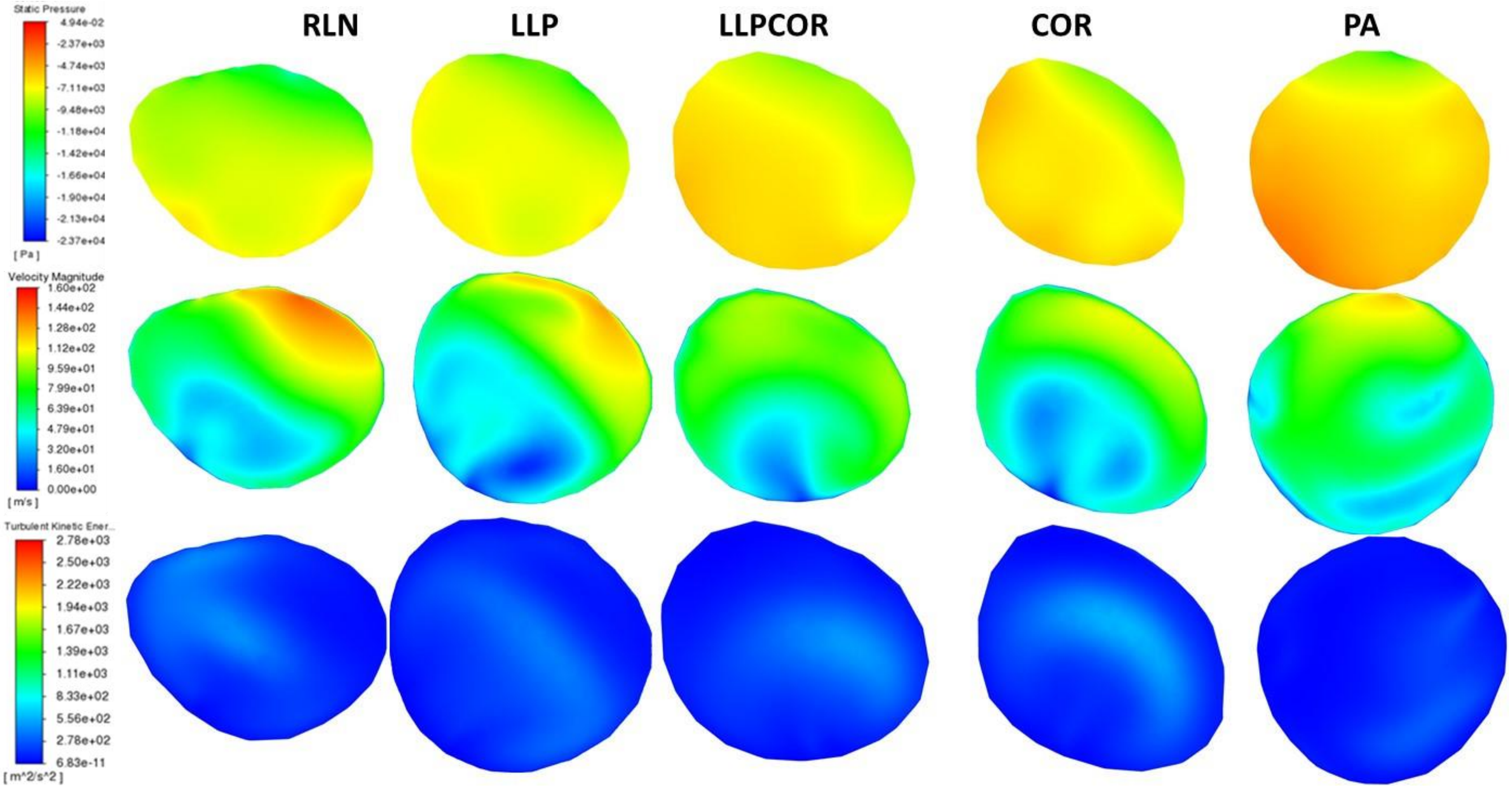

Figure H Pressure (top row), velocity (middle row), and turbulent kinetic energy (bottom row) for the sagittal sections by procedure. The colors correspond to the respective scales in Figure 5.5A.

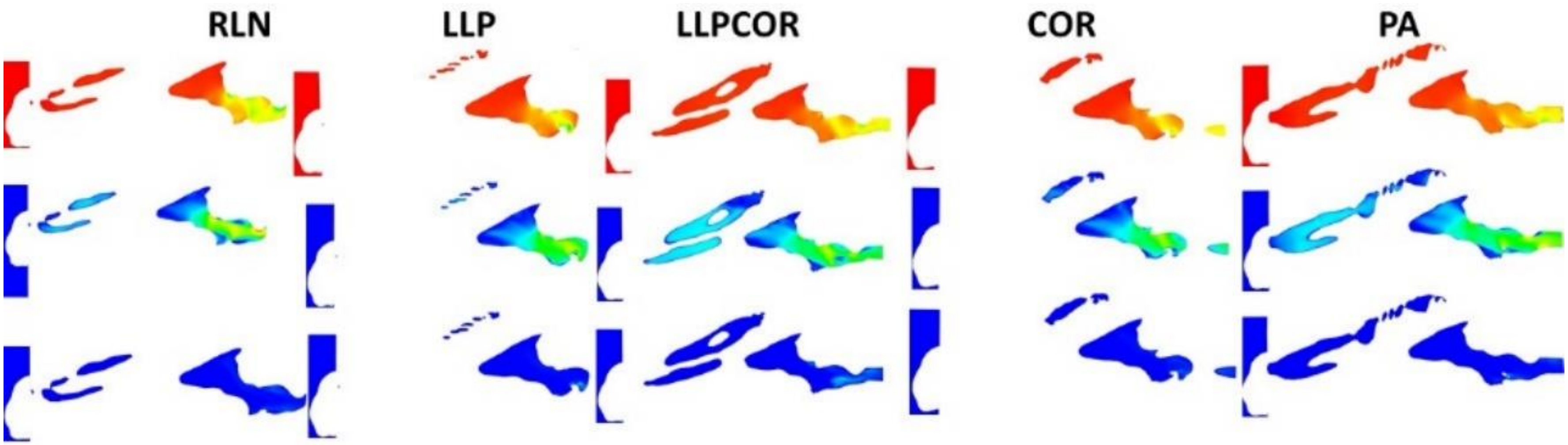

Figure I Pressure (top row), velocity (middle row), and turbulent kinetic energy (bottom row) for the sagittal sections by procedure. These are enlarged versions of Figure 5.5H, looking specifically at the larynx. The colors correspond to the respective scales in Figure 5.5A.

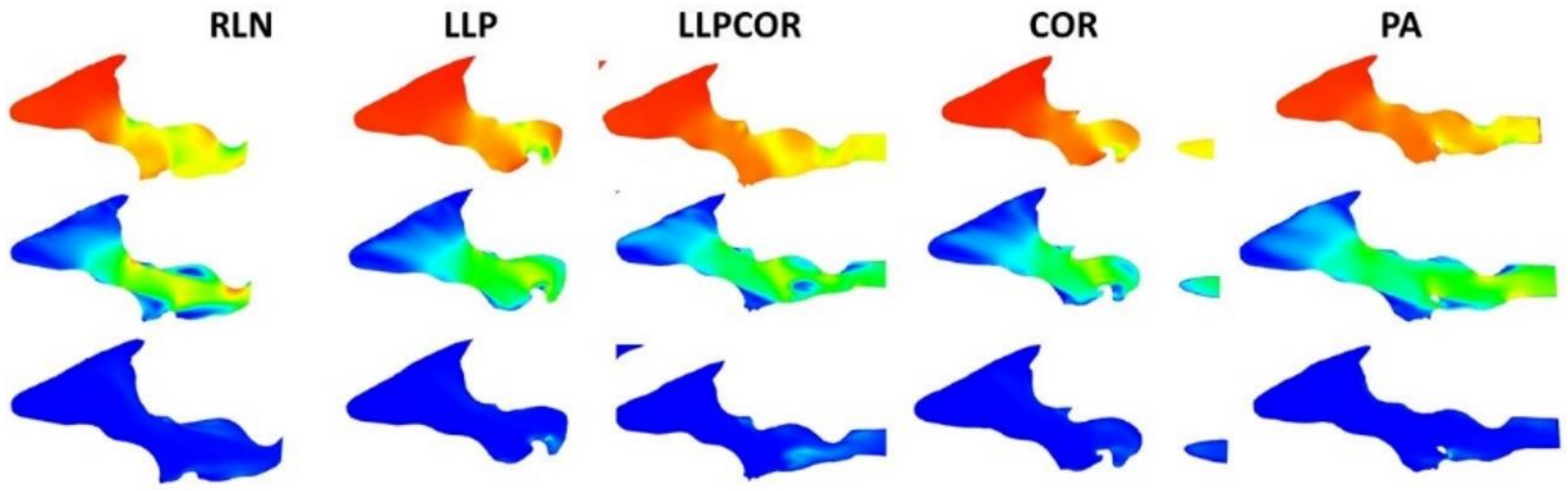

Figure J Pressure (top row), velocity (middle row), and turbulent kinetic energy (bottom row) for the parasagittal sections by procedure. The top three rows are the left parasagittal plane; the bottom three rows are the right parasagittal plane. The colors correspond to the respective scales in Figure 5.5A.

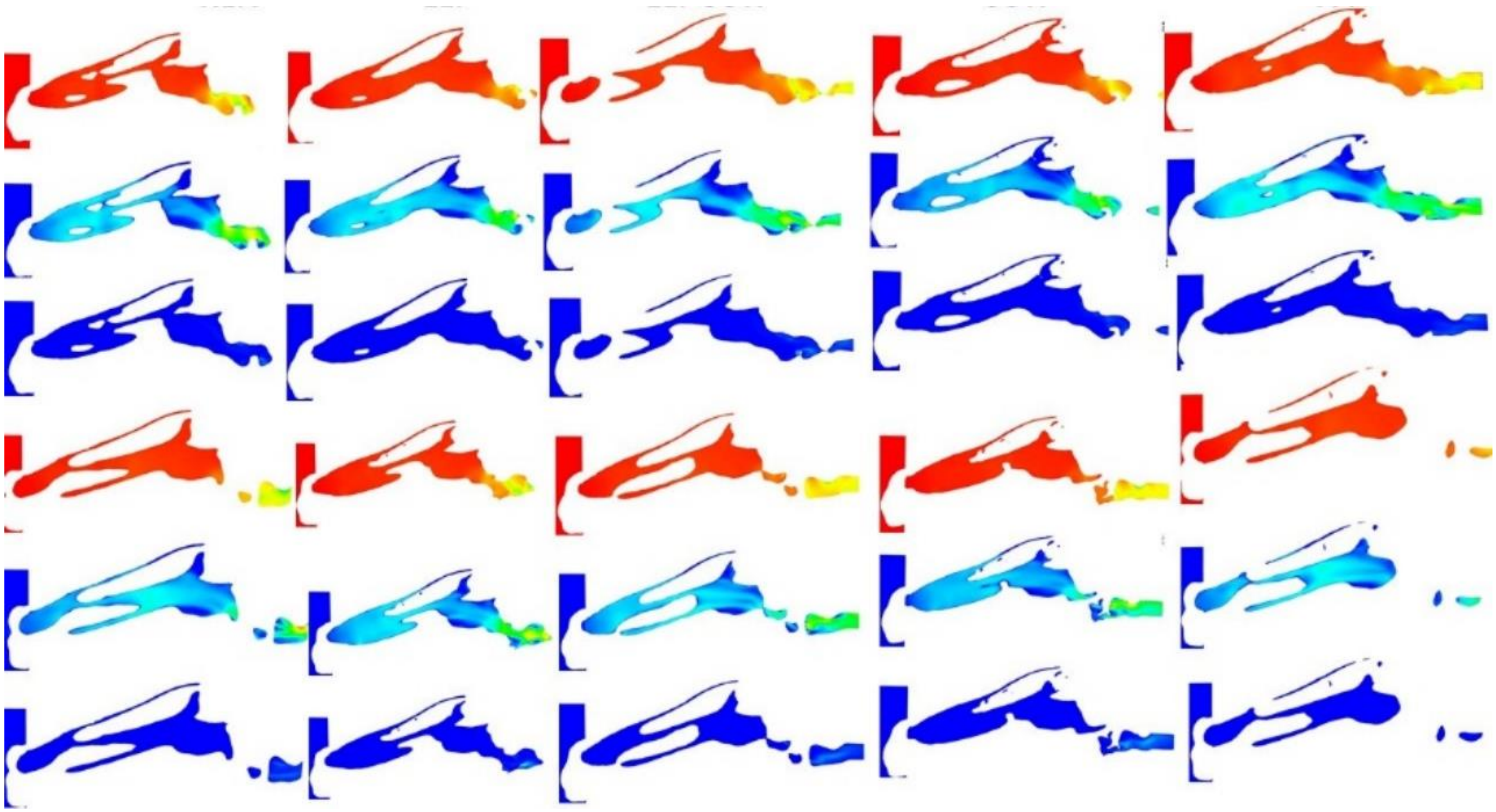

Figure K Pressure (top row), velocity (middle row), and turbulent kinetic energy (bottom row) for the parasagittal sections by procedure. The top three rows are the left parasagittal plane; the bottom three rows are the right parasagittal plane. These are enlarged versions of the same planes as Figure 5.5J, focused on the larynx. The colors correspond to the respective scales in Figure 5.5A.

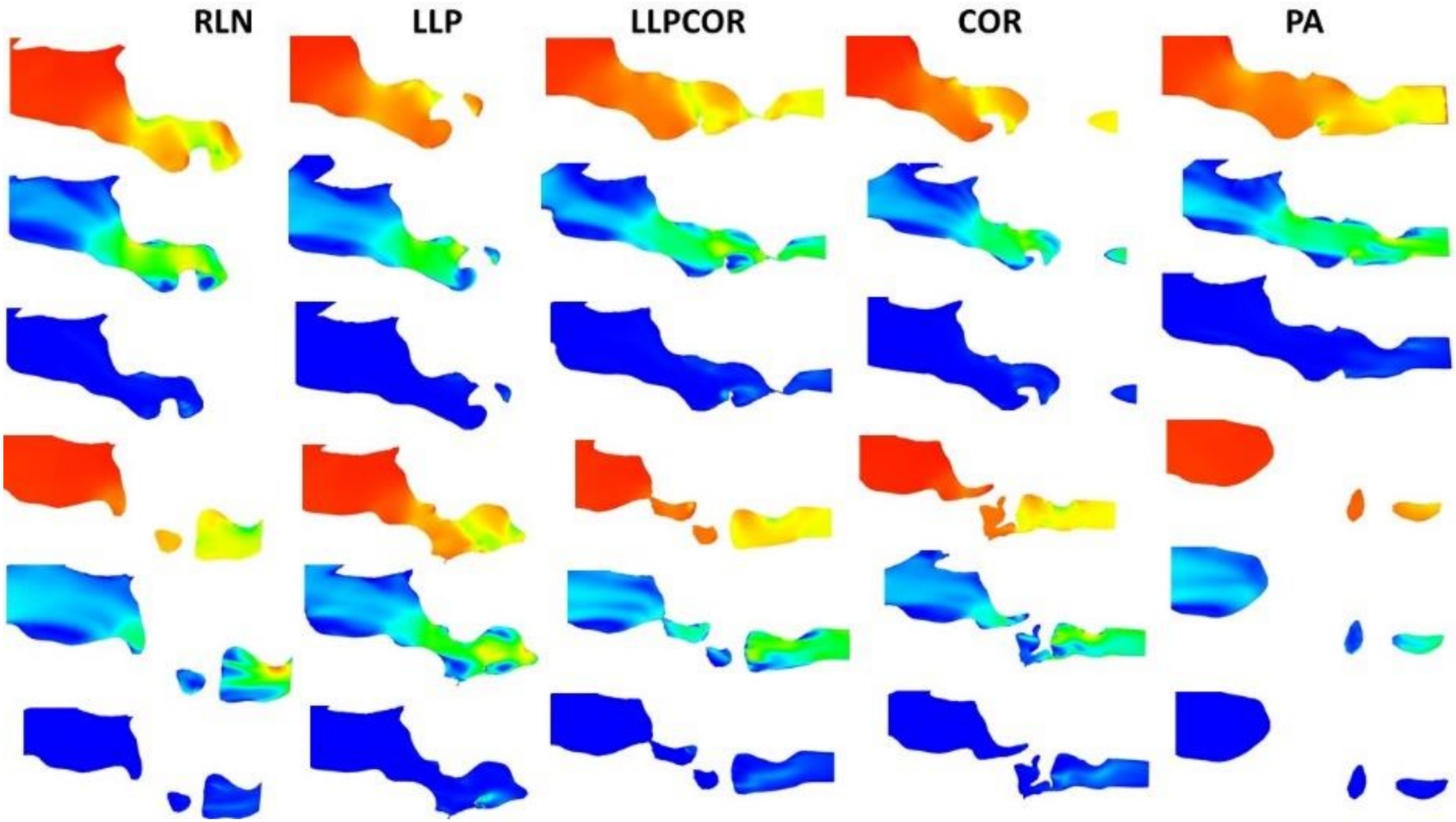

Figure L Pressure (top row), velocity (middle row), and turbulent kinetic energy (bottom row) for the transverse sections by procedure. The top three rows are the mid-nostril transverse plane; the bottom three rows are the mid-pharyngeal transverse plane. The colors correspond to the respective scales in Figure 5.5A.

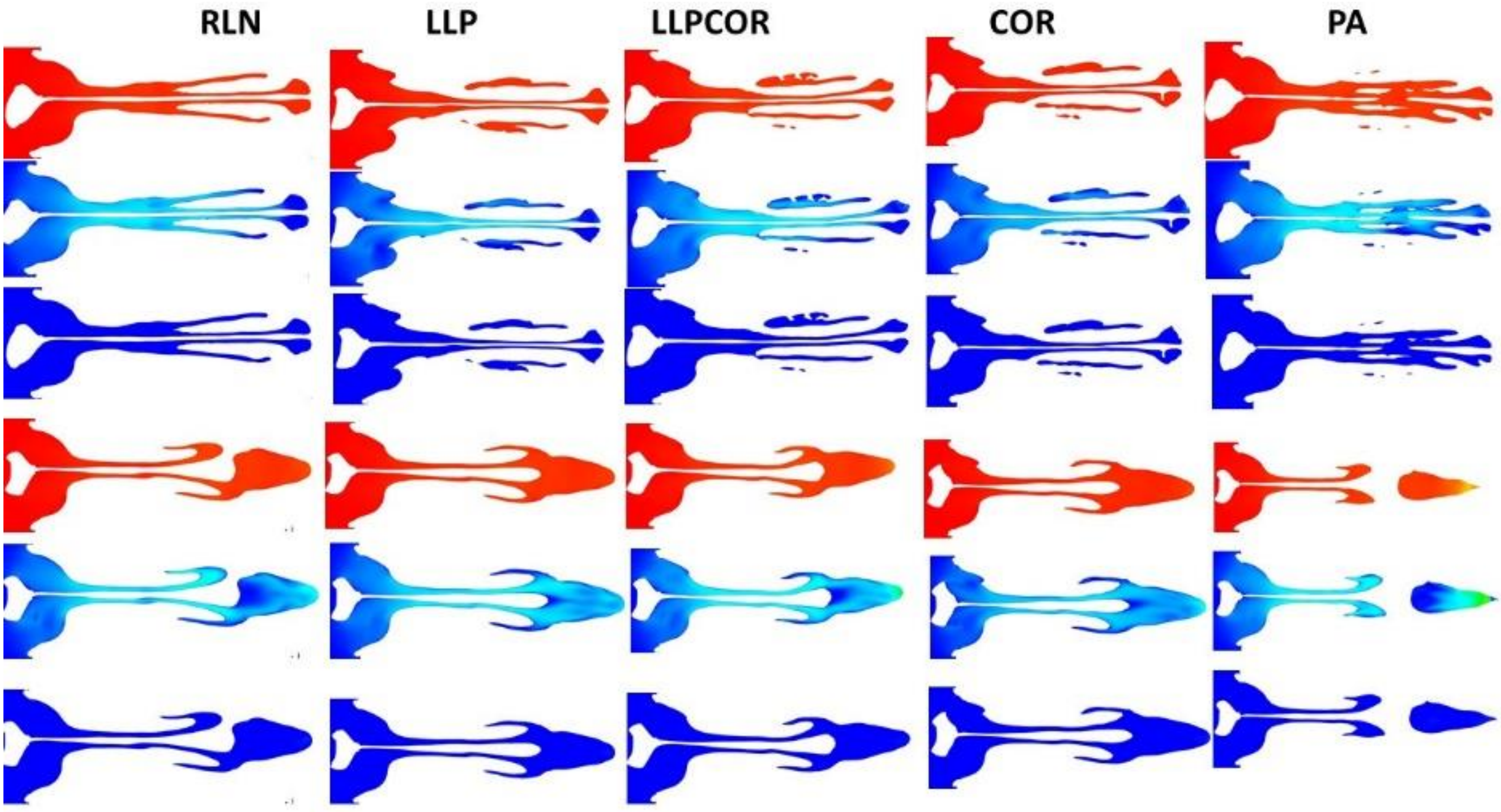

Figure M Pressure (top row), velocity (middle row), and turbulent kinetic energy (bottom row) for the dorsal laryngeal transverse sections by procedure. The colors correspond to the respective scales in Figure 5.5A.

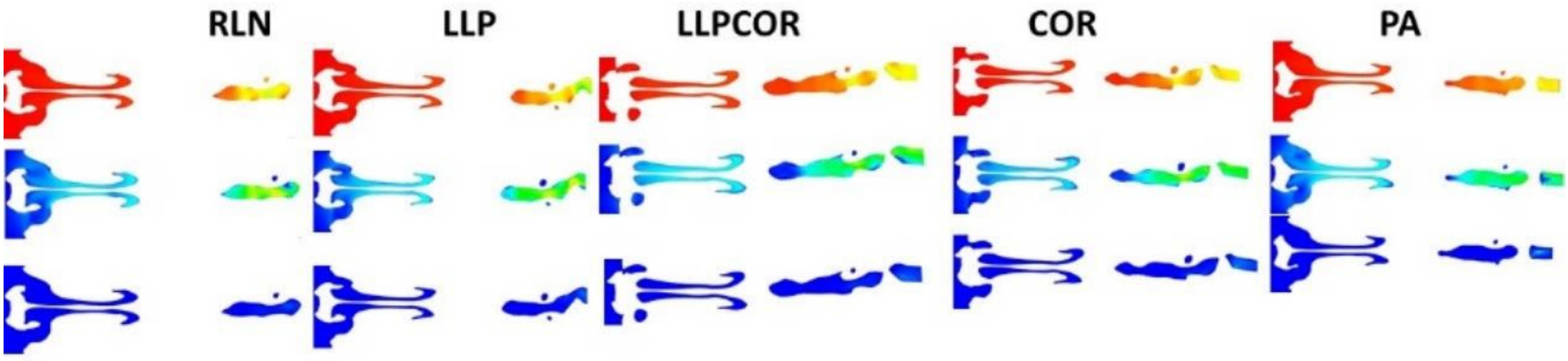

Figure N Pressure (top row), velocity (middle row), and turbulent kinetic energy (bottom row) for the dorsal laryngeal transverse sections by procedure. These are enlarged versions of the same planes as Figure 5.5M, focused on the larynx. The colors correspond to the respective scales in Figure 5.5A.

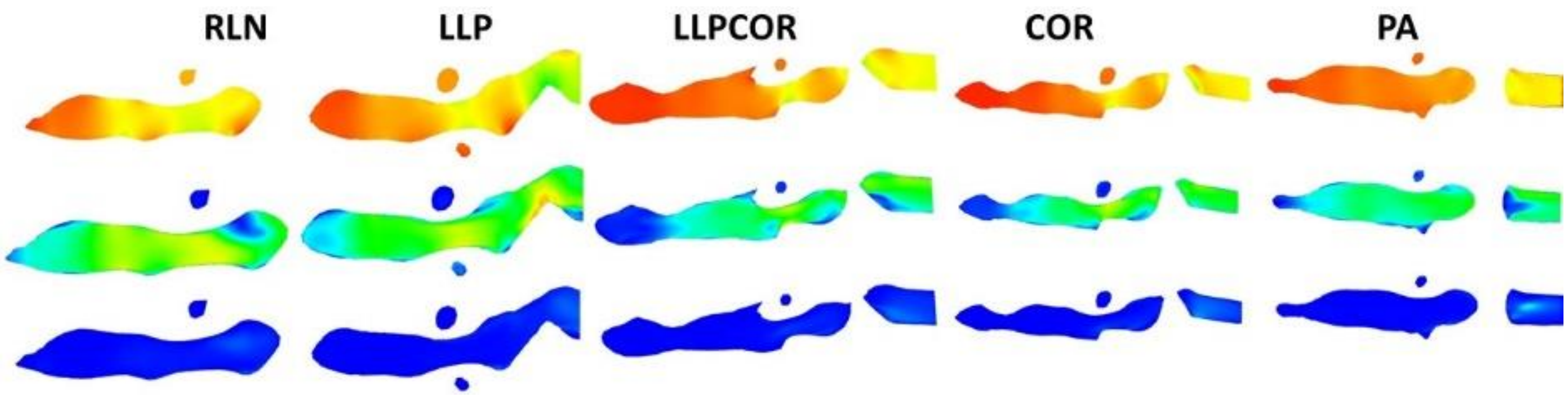

Figure O Pressure (top row), velocity (middle row), and turbulent kinetic energy (bottom row) for the transverse sections by procedure. The top three rows are the mid-laryngeal transverse plane; the bottom three rows are the ventral laryngeal transverse plane. The colors correspond to the respective scales in Figure 5.5A.

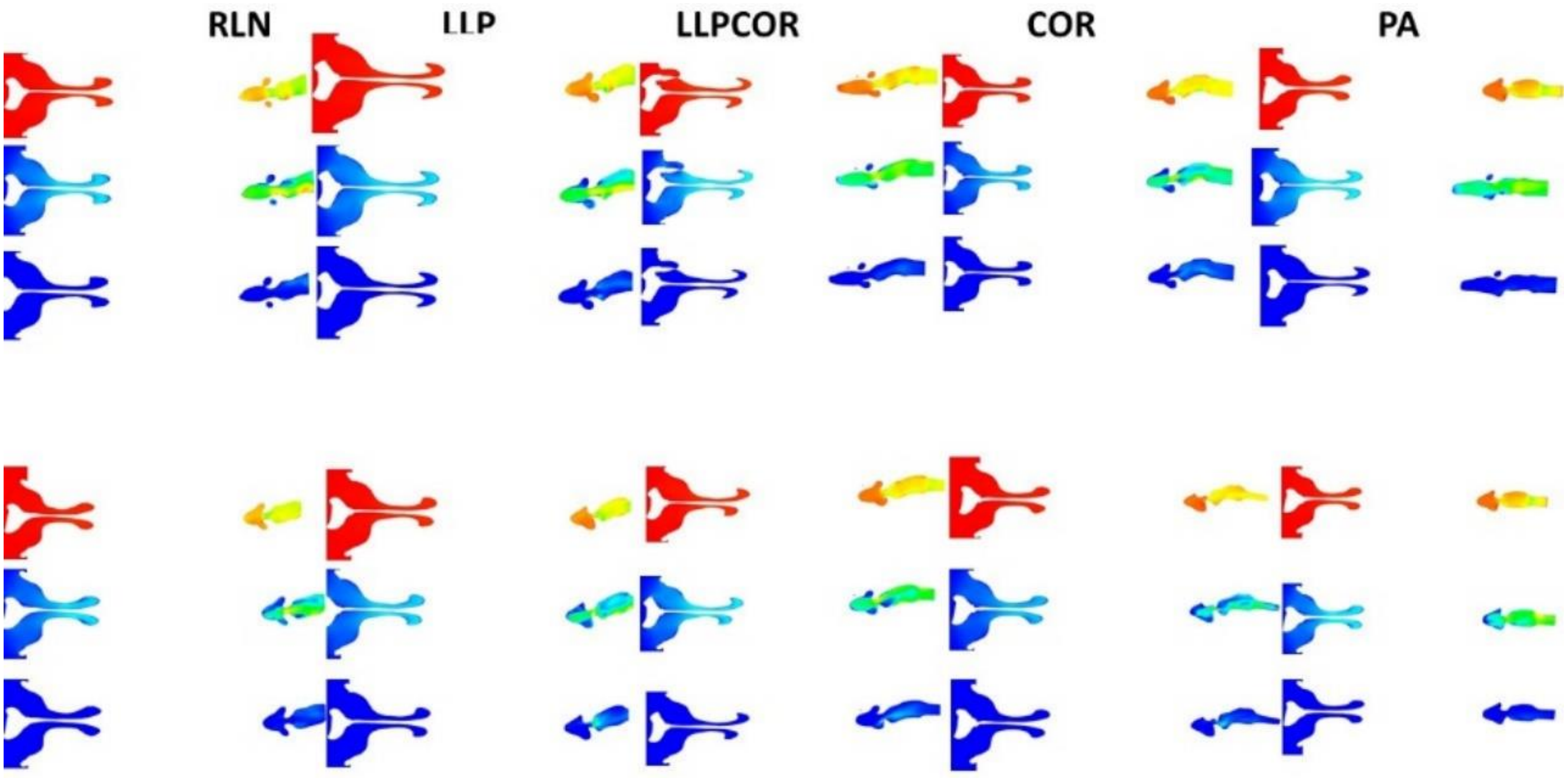

Figure P Pressure (top row), velocity (middle row), and turbulent kinetic energy (bottom row) for the transverse sections by procedure. The top three rows are the mid-laryngeal transverse plane; the bottom three rows are the ventral laryngeal plane. These are enlarged versions of the same planes as Figure 5.5O, focused on the larynx. The colors correspond to the respective scales in Figure 5.5A.

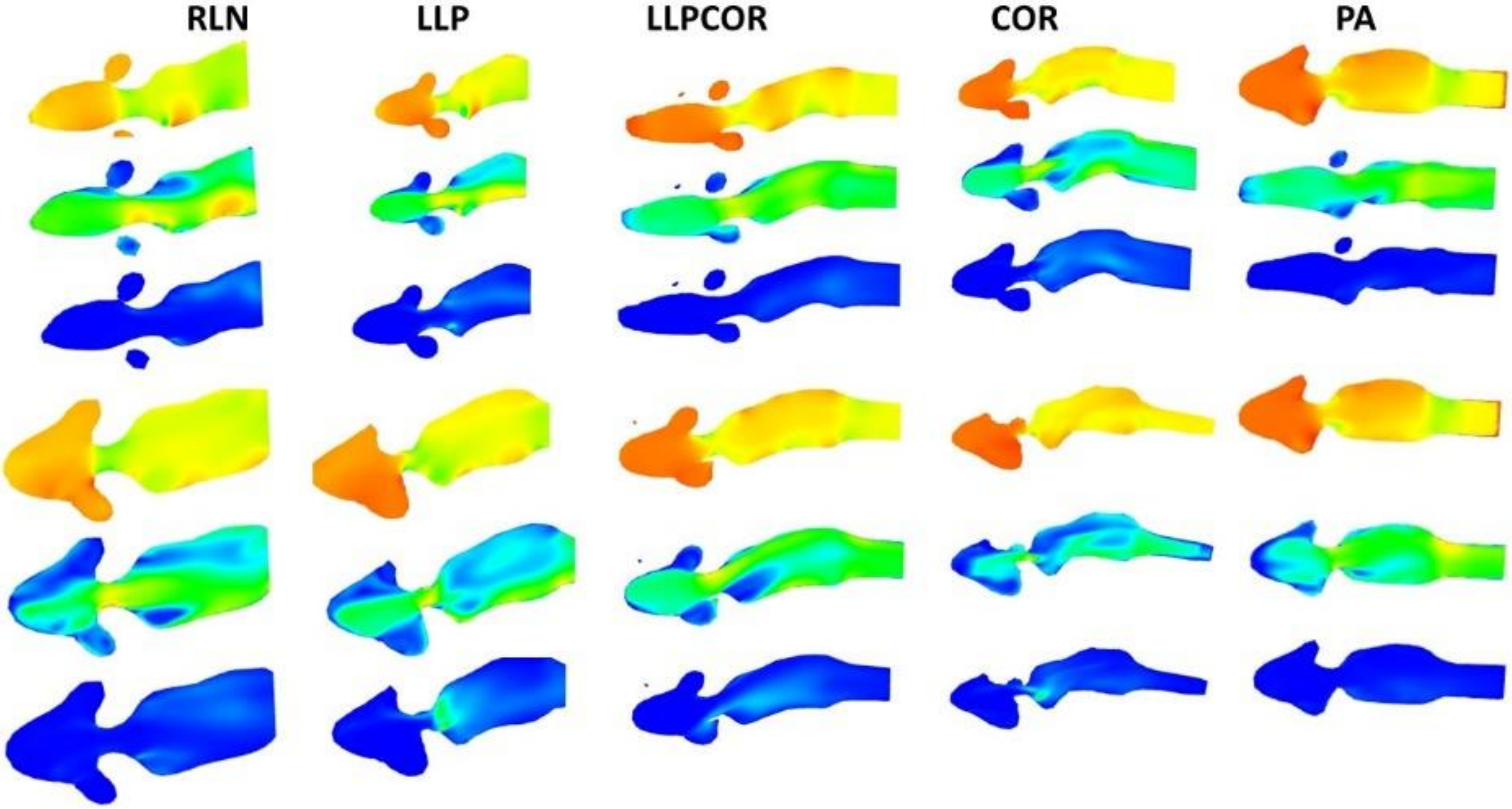

Supplement: Supplementary file 1 [file Data_Sheet_1.pdf]
